# 4443 Tailoring Professional Development to CTS Trainees

**DOI:** 10.1017/cts.2020.223

**Published:** 2020-07-29

**Authors:** Megan Maxwell, Elizabeth Hexner, Rachel McGarrigle, Emma Meagher

**Affiliations:** 1 University of Pennsylvania School of Medicine

## Abstract

OBJECTIVES/GOALS: Penn instituted a Professional Development Core (PDC) to complement existing CTS education programs. Sessions were designed to advance participant knowledge and skills in key competency areas including communication, expectation setting, implicit bias and organizational structure, self-efficacy and resilience in order to enhance abilities to successfully execute career and research goals. METHODS/STUDY POPULATION: The PDC enrolled 4 cohorts totaling 87 trainees and scholars from 2016-2019. This included 35% predoctoral trainees (27 MD, 3 PhD), 39% postdoctoral trainees (29 MD, 3 PhD, 2 VMD/DVM), and 26% junior faculty (16 MD, 6 MD/PhD, 1 PhD). The two-year curriculum consists of 14 lunchtime sessions held bimonthly during academic terms. Session structures include a variety of interactive presentations, activities and facilitated discussions as well as reading material, assessment tools, and case studies. Facilitators include topic experts in academia, entrepreneurship, communications, and professional and personal development. The program is evaluated qualitatively through student satisfaction surveys after each session. RESULTS/ANTICIPATED RESULTS: Of the 2018-2019 participants, 90.8% rated the overall quality of PDC sessions as Very Good (56.05%) or Outstanding (34.75%). DISCUSSION/SIGNIFICANCE OF IMPACT: Feedback indicates that the group benefited from combining predoctoral and postdoctoral trainees, although not all content was immediately relevant to early stage trainees. Trainees appreciated the opportunity to engage with experts in disciplines typically considered outside of traditional science but critical to CTS career success. The flexibility of the curriculum allowed for inclusion of timely topics, newer suites of sessions focus on the multiple dimensions of valuing your science.

